# Site-specific modification of ED-B-targeting antibody using intein-fusion technology

**DOI:** 10.1186/1472-6750-11-76

**Published:** 2011-07-21

**Authors:** Sina Möhlmann, Peter Bringmann, Simone Greven, Axel Harrenga

**Affiliations:** 1 Bayer Healthcare Research Center, Aprather Weg 18a, 42113 Wuppertal, Germany; 2 Bayer HealthCare LLC, 455 Mission Bay Blvd South, San Francisco, CA 94158, USA; 3 Bayer Healthcare, Nattermannallee 1, 50829 Cologne, Germany

## Abstract

**Background:**

A promising new approach in cancer therapy is the use of tumor specific antibodies coupled to cytotoxic agents. Currently these immunoconjugates are prepared by rather unspecific coupling chemistries, resulting in heterogeneous products. As the drug load is a key parameter for the antitumor activity, site-specific strategies are desired. Expressed protein ligation (EPL) and protein trans-splicing (PTS) are methods for the specific C-terminal modification of a target protein. Both include the expression as an intein fusion protein, followed by the exchange of the intein for a functionalized moiety.

**Results:**

A full-length IgG specific for fibronectin ED-B was expressed as fusion protein with an intein (*Mxe *GyrA or *Npu *DnaE) attached to each heavy chain. In vitro protocols were established to site-specifically modify the antibodies in high yields by EPL or PTS, respectively. Although reducing conditions had to be employed during the process, the integrity or affinity of the antibody was not affected. The protocols were used to prepare immunoconjugates containing two biotin molecules per antibody, attached to the C-termini of the heavy chains.

**Conclusion:**

Full-length antibodies can be efficiently and site-specifically modified at the C-termini of their heavy chains by intein-fusion technologies. The described protocols can be used to prepare immunoconjugates of high homogeneity and with a defined drug load of two. The attachment to the C-termini is expected to retain the affinity and effector functions of the antibodies.

## Background

Monoclonal antibodies have been approved as therapeutic agents for indications including viral infections, immunological disorders, transplant rejection and cancer [1]. They often act by blocking the function of their target molecule. More demanding is the therapy of cancer by antibodies requiring the specific recognition and subsequent elimination of tumor cells.

Several mechanisms have been described how therapeutic antibodies elicit cell death, including the triggering of apoptosis and the recruitment of the immune system. While therapeutic antibodies have been approved working by these mechanisms (e.g. Rituximab [2], Trastuzumab [3], Alemtuzumab [4]) their cytotoxic potential is generally not sufficient to completely eliminate the malignant cells. Higher efficacies have been observed if the antibody is coupled to toxic agents like radioisotopes (radioimmunoconjugates) or chemical drugs (antibody-drug-conjugates, ADC) [5]. Several of these conjugates have been approved for cancer (Ibritomomab, Tositumomab) or are in clinical development (e.g. Trastuzumab-DM1).

Coupling of toxic agents to therapeutic antibodies also paves the way for new tumor associated antigens as these are not required to be present on the surface of the malignant cells. An example is the extra domain B (ED-B) of fibronectin, a protein of the extracellular matrix. ED-B-containing fibronectin is a splice variant associated with angiogenesis and tissue remodeling [6]. High levels of ED-B expression have been detected in most solid tumors and in vivo studies with ED-B specific monoclonal antibody formats show the selective accumulation in tumors and metastases. Accordingly, ED-B is a promising target for antibody-based cancer treatment [7,8] and the results of first clinical trials with ED-B specific antibody fragment conjugates are encouraging [9,10].

Current methods for the preparation of immunoconjugates rely on the chemical coupling to lysine, cysteine or tyrosine side chains [11]. These methods are rather unspecific and result in heterogeneous products. As the drug load - number of toxophore per antibody - is a key parameter for the antitumor activity of immunoconjugates [12-14] more site-specific coupling reactions are desired. Approaches employing the carbohydrate moieties [15,16], the N- and the C-terminus [17,18] of full-length IgG antibodies have been described. However, the carbohydrates are important for the effector functions of the Fc domain [19] and the N-terminus of antibodies is close to their antigen binding site which may result in decreased affinity after modification. This leads to the C-terminus as a preferred site for specific drug attachment.

Several enzymatic approaches have been described for the modification of protein C termini [20]. They have in common that the target protein is expressed in fusion with a C-terminal tag containing the modification site. A common drawback of these methods is an incomplete conversion. Without the possibility for separation, this would result in heterogeneous preparations of low averaged drug loads. Interestingly, the intein tag is cleaved off from the target protein during modification, facilitating preparative separation of modified from non-modified protein. Inteins encompass catalytic domains which lead to the formation of a thioester bond at their junction to the target protein. This thioester bond can be employed to exchange the intein for a C-terminal probe. The probe is eventually connected via a native peptide bond [21].

Methods for intein-mediated C-terminal protein modification encompass expressed protein ligation (EPL) and protein trans-splicing (PTS). In EPL, the target protein is fused to a modified full-length intein. The intein is cleaved off by the addition of a thiol reagent, leaving a thioester bond (first step), and the target protein is ligated to a probe functionalized with an N-terminal cysteine residue (second step) [22,23]. In PTS, inteins are used which are split into two parts with high affinity to each other. The large N-terminal part is fused to the target protein. The probe is functionalized with the small C-terminal part of the intein. Their combination results in a functional intein, which splices itself out and concomitantly fuses the target protein to the probe [24-26].

**Figure 1:**
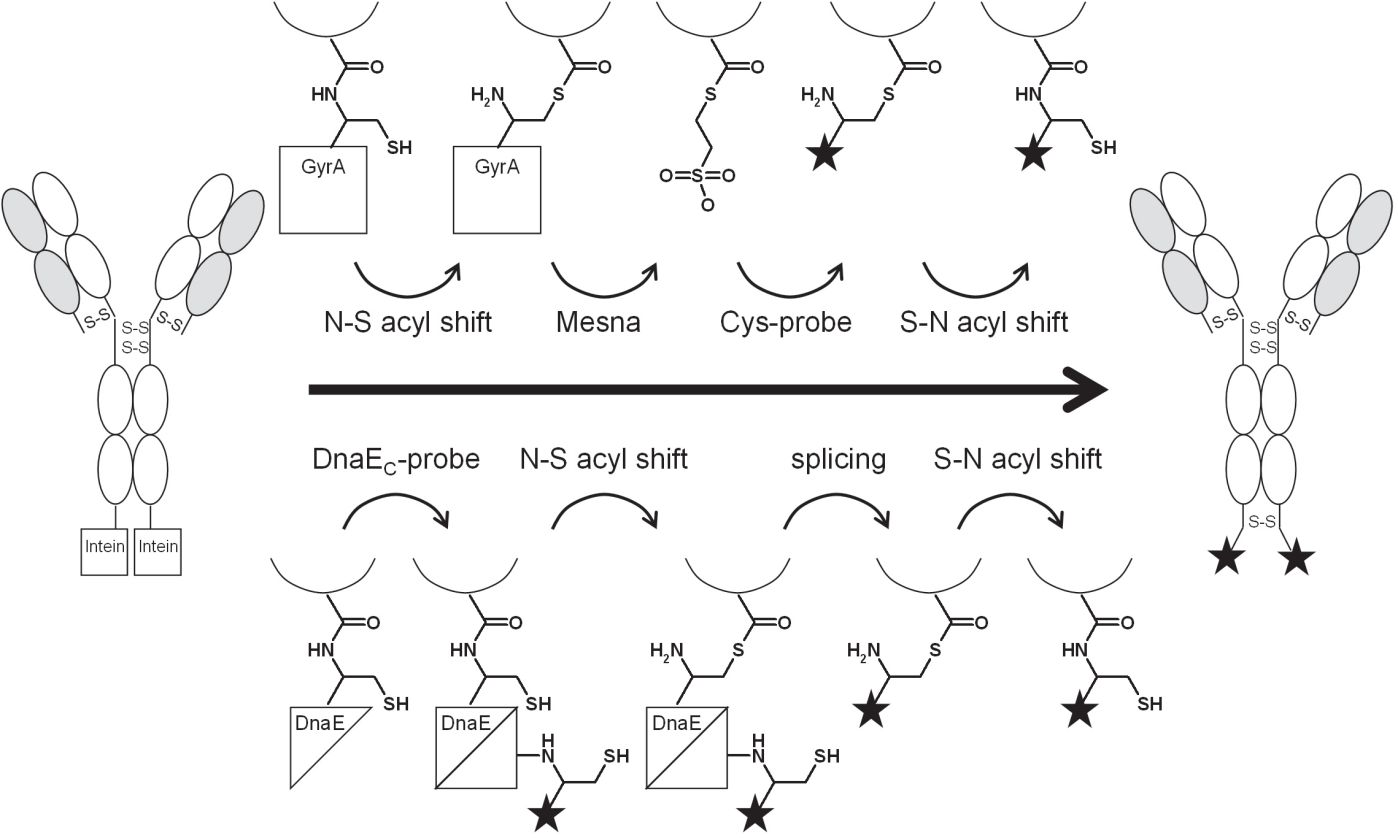
**Illustration of chemical steps during in vitro processing of IgG-intein fusion protein**. Above the arrow, the chemistry of GyrA intein is shown. After intein-catalyzed N-S acyl shift, GyrA is substituted by Mesna. Mesna-thioester is cleaved by the probe functionalized with an N-terminal cysteine residue, and finally rearranges to a stable peptide bond. Below the arrow, the chemistry of DnaE intein is shown. The intein has to be activated by association of its N- and C-terminal part. The probe is attached to the C-terminal part and transferred after the intein-catalyzed N-S acyl shift.

Intein-fusion technologies have already been used for several applications, including the derivatization of small single-chain and single-domain antibody formats with fluorophores and micelles, respectively [27-29]. Here we explored the use of two distinct inteins for C-terminal modification of a full-length antibody. The first is the GyrA intein from *Mycobacterium xenopi *(*Mxe*) which is a 'minimal' intein of 198 residues [30]. It can be used for protein modification by EPL [31]. The second is the split DnaE intein from *Nostoc punctiforme *(*Npu*) [32,33]. It consists of an N-terminal part of 102 residues and a C-terminal of 36 residues. *Npu *DnaE was employed for protein modification by PTS. Each intein was fused to the heavy chain of anti ED-B IgG1, resulting in two inteins per antibody (Figure 1). The fusion proteins were expressed by secretion from transfected human embryonic kidney (HEK) cells. After purification, in vitro cleavage by *Mxe *GyrA or trans-splicing by *Npu *DnaE yielded 60% or 80%, respectively, of completely processed antibody. The protocols described resulted in functional antibodies and were used to generate immunoconjugates containing two biotin moieties per protein molecule, attached site-specifically to the C-termini of the heavy chains.

## Results and Discussion

### Production of L19 IgG antibodies with C-terminal intein domains

Antibodies require an oxidizing environment for their expression as they contain several disulfide bonds important for proper folding. In addition, glycosylation has been shown to be important for the effector functions of antibodies [19]. They are therefore usually produced by mammalian expression systems. We chose transiently transfected HEK293-6E cells for the expression of L19 IgG antibody which is specific for the ED-B domain of fibronectin. Endogenous signal sequences ensure the secretion into the extracellular medium and therefore the proper folding of the immunoglobulin domains. Connected via an 11 residue linker, the heavy chain was fused to an intein domain. We tested two distinct inteins, GyrA from *Mycobacterium xenopi *[31] and the split intein DnaE from *Nostoc punctiforme *[32]. In case of DnaE, L19 IgG was fused to the N-terminal domain DnaE_N _(residues 1-102). Both inteins were extended C-terminally by a decahistidine tag for purification purposes.

**Figure 2:**
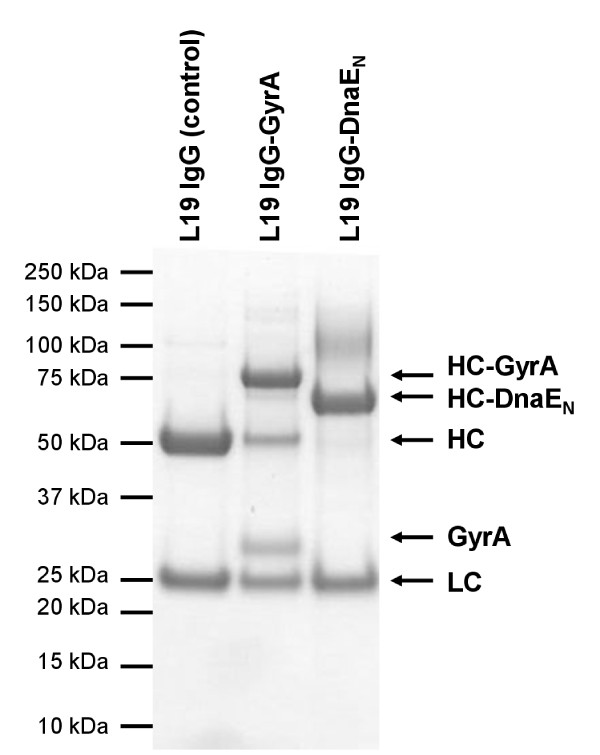
**In vivo cleavage**. Mammalian expression medium was conducted to His tag affinity chromatography and the eluate was analyzed by reducing SDS-PAGE (Coomassie stained). 10-15% of L19 IgG-GyrA is cleaved during expression. L19 IgG-DnaE_N _shows no in vivo cleavage (HC: heavy chain, LC: light chain).

Antibody-intein fusion proteins were obtained in good yields (90-110 mg/L). They were purified by His tag affinity chromatography and subsequent gel filtration. A common problem with GyrA fusion proteins is the occurence of intein cleavage during expression. This in vivo cleavage decreases the product yield and is detectable by the co-purification of the separated intein domain after His Tag affinity chromatography (Figure 2). However, judged by the subsequent gel filtration elution profile (not shown), only 10-15% in vivo cleavage was observed for GyrA. DnaE_N _showed no in vivo cleavage as expected for a split intein.

### Preparation of immunoconjugates by *Mxe *GyrA intein mediated EPL

In vitro cleavage of L19 IgG-GyrA fusion protein was induced by the addition of 50 mM Mesna which has been reported to form a long-living thioester at the C-terminus. This thioester can be employed to link to the IgG a probe that is functionalized with an N-terminal cysteine residue. It is added concomitantly with Mesna but in lower concentration (5 mM). The cysteine replaces the Mesna-thioester, resulting in a stable peptide bond between the IgG and the probe (Figure 1).

**Figure 3:**
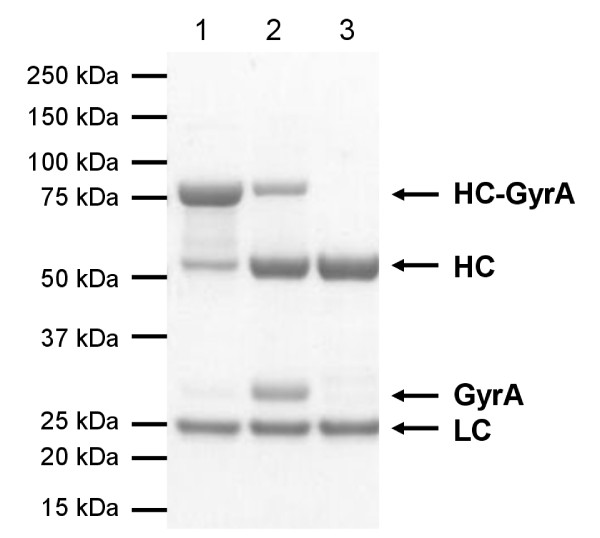
**L19 IgG-GyrA after EPL**. Analysis by reducing SDS-PAGE (Coomassie stained). Purified fusion protein was treated with 50 mM Mesna and 5 mM cysteine for 22h at RT. After dialysis, cleaved L19 IgG was purified by His tag affinity chromatography (1: L19 IgG-GyrA before cleavage, 2: after cleavage, before purification, 3: after purification, HC: heavy chain, LC: light chain).

**Figure 4:**
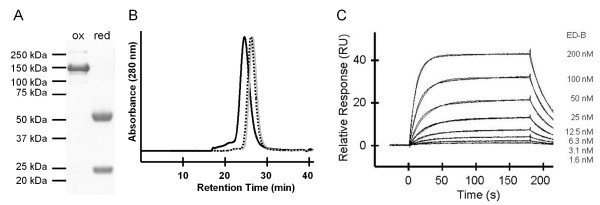
**Analysis of L19 IgG-GyrA integrity after EPL**. (A) Non-reducing (ox) and reducing (red) SDS-PAGE (Coomassie stained). (B) Analytical Gel Filtration. Black: L19 IgG-GyrA before cleavage. Dotted: Purified EPL product. Grey: L19 IgG control antibody. (C) SPR analysis. Results were fitted to a one-site-binding model with a K_D _of 91.4 nM.

To study the cleavage efficiency of the L19 IgG-GyrA fusion protein and to test the antibody integrity after EPL, cysteine was used as 'probe'. After 22h, Mesna and cysteine were removed by intensive dialysis to regenerate and stabilize the disulfide bonds of the antibody. L19 IgG cleaved on both heavy chains could be separated from non- or partly-cleaved antibody by His Tag affinity chromatography (Figure 3). The yield of completely cleaved antibody was about 60%. To test if its functionality had been damaged by the reducing conditions used during EPL it was intensively analyzed. SDS-PAGE under non-reducing conditions resulted in a single band at 150 kDa (calculated: 146.3 kDa), showing that interchain disulfide bonds were intact (Figure 4A). Analytical gel filtration confirmed the integrity of the antibody, eluting in a single peak very similar to unmodified L19 IgG (control antibody) (Figure 4B). Finally, interaction studies by Surface Plasmon Resonance (SPR) were employed to evaluate antibody functionality after EPL. The antigen binding part was analyzed by investigation of the interaction with the ED-B domain. Binding kinetics and the dissociation constant (~90 nM) were the same as for the L19 IgG control antibody (Figure 4C). To test the functionality of the Fc part, the interaction with the high affinity Fcγ RI/CD64 and with the low affinity Fcγ RIIIA/CD16a was analyzed by SPR. Binding kinetics and the dissociation constants for both Fcγ receptors (Fcγ RI/CD64: ~0.2 nM; Fcγ RIIIA/CD16a: ~60nM) did not change significantly demonstrating preservation of full functionality (see Additional file 1). In summary, these results show that antigen binding as well as effector functions of L19 IgG have not been affected by the EPL process.

**Figure 5:**
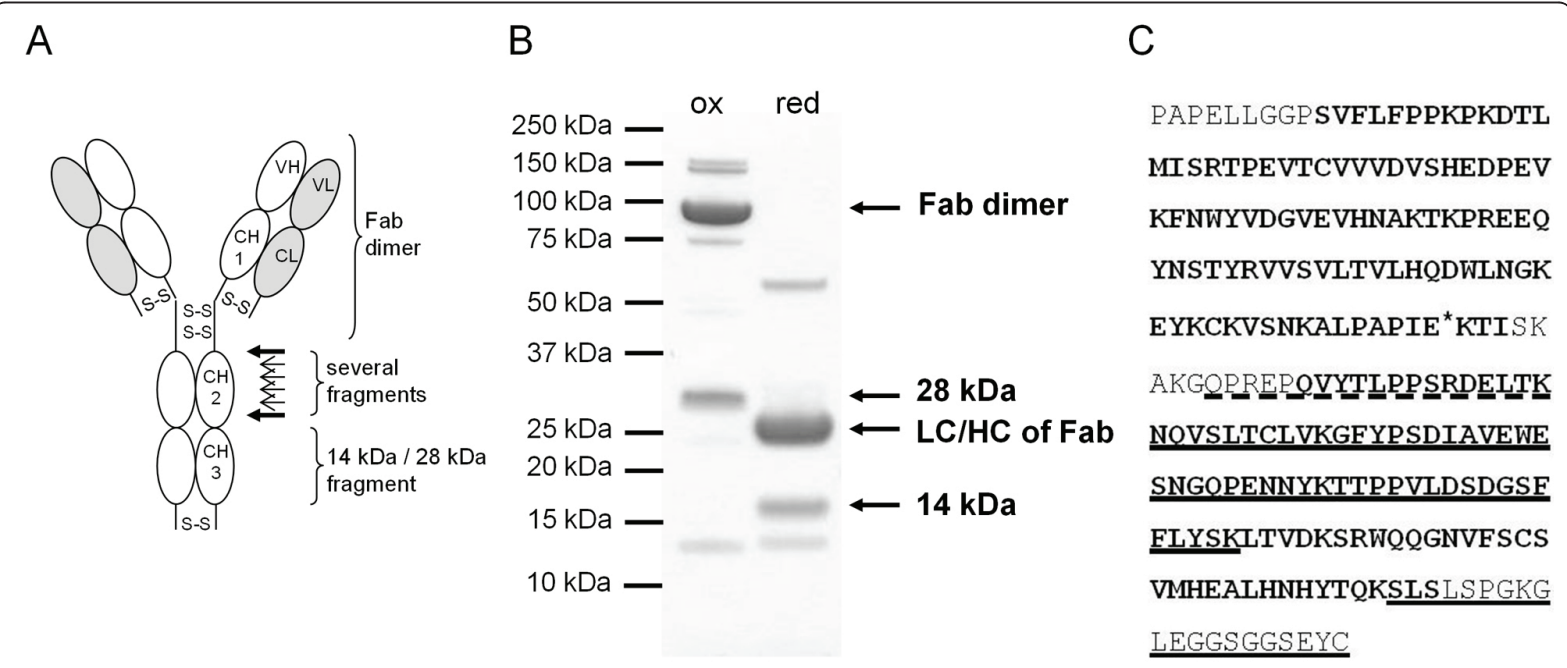
**High yield of cysteine derivatization after L19 IgG-GyrA in vitro cleavage**. Purified EPL product was pepsin digested. (A) Scheme indicating observed pepsin cleavage sites. (B) SDS-PAGE analysis (Coomassie stained). Non-reducing (ox) conditions show the Fab dimer and a prominent 28 kDa band. After reduction (red), a 14 kDa band is observed. (C) Mass spectrometry analysis results, illustrated by the amino acid sequence of L19 IgG Fc domain. A sample after pepsin digest was analyzed by ESI-TOF. Two prominent masses were found: The Fab dimer (calculated: 96061.5, observed: 96061.2 Da) and a fragment (calculated: 27857.1 Da, observed: 27856.3 Da) corresponding to a disulfide connected CH3 domain cleaved at the indicated site (asterisk). Sequence analysis was performed by tryptic in-gel digest of the 28 kDa and 14 kDa band in (B) followed by ESI-Q-TOF. Only sequences of the CH3 domain were observed. Bold: Immunoglobuline domains (CH2 and CH3). Underlined: Sequences found in the 28 kDa and 14 kDa band. Dashed underlined: Sequences only found in the 14 kDa band.

A side reaction of EPL is the hydrolysis of the thioester instead of its derivatization with cysteine. To estimate the yield of L19 IgG-Cys, a pepsin digest assay was employed. Pepsin can be used to prepare Fab dimers from an IgG1 as it cleaves C-terminal to the interchain disulfide bonds connecting the heavy chains [34]. The Fc part of the antibody is cleaved into several fragments. Mass spectrometry analysis of L19 IgG control antibody after pepsin digest (pH 4.5) revealed that the most C-terminal cleavage site is the linker between CH2 and CH3 domain, leaving the CH3 domain intact (Figure 5A). This could be used for the observation of cysteine derivatization: If cysteines are attached C-terminal to the heavy chains they are expected to form an interchain disulfide bond, resulting in a 28 kDa fragment under non-reducing conditions. However, if one or both cysteines are missing, 14 kDa fragments are observed. Non-reducing SDS-PAGE after pepsin digest of L19 IgG-Cys revealed the occurrence of a 28 kDa band which was replaced by a 14 kDa band after reduction (Figure 5B). Both bands were excised from the gel and analyzed after tryptic digest with HPLC ESI-Q-TOF. Clearly, only peptides from the CH3 domain were found (Figure 5C). Also the C-terminal peptide was observed, carrying the cysteine. Interestingly, the 14 kDa band is hardly detectable on the non-reducing SDS-PAGE gel. Mass spectrometry analysis of a non-reduced sample after pepsin digest confirmed this observation: We detected a 28 kDa fragment but no 14 kDa fragment (Figure 5C). We conclude that the fraction of hydrolyzed protein is minor and that cysteine derivatized L19 IgG was obtained in high yield. This would result in a homogeneous drug load of two. In addition, a C-terminal disulfide bond is readily formed. This may stabilize the immunoconjugate and hinders undesired side reactions of thiol groups in the oxidizing environment antibodies are held.

**Figure 6:**
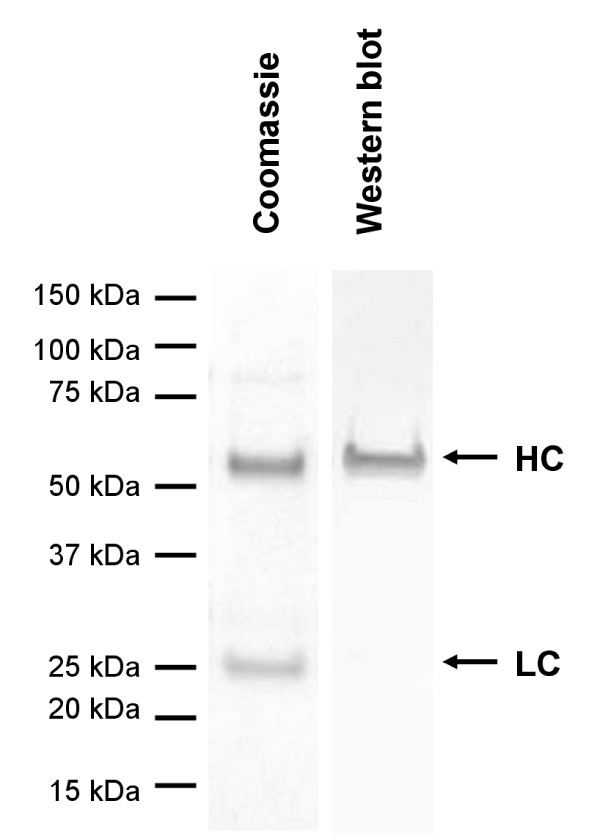
**Biotinylation of L19 IgG-GyrA after in vitro cleavage**. EPL was performed with 50 mM Mesna and 5 mM CysLys-Biotin, the product was purified by His tag affinity chromatography and analyzed by reducing SDS-PAGE. Western blotting with Streptavidin-Alkaline-Phosphatase revealed specific biotin derivatization of the heavy chain.

The aim of our work was to use GyrA intein for the attachment of probes functionalized with an N-terminal cysteine residue. To show the usability of the protocol for this purpose, a CysLys-Biotin peptide was added instead of cysteine. After SDS-PAGE and Western blotting, the specific attachment of biotin to the heavy chain was verified by Streptavidin-based staining (Figure 6). In summary, the described EPL protocol is applicable for the site specific modification of full-length IgG by probes attached to an N-terminal cysteine residue.

### Preparation of immunoconjugates by *Npu *DnaE intein catalyzed PTS

L19 IgG-DnaE_N _contains an incomplete intein which was activated in vitro by the addition of a peptide comprising the missing C-terminal 36 residues, followed by the naturally occurring post-intein sequence CFN. This DnaE_C _peptide can be employed to transfer a C-terminally attached probe to L19 IgG by PTS (Figure 1). Importantly, trans-splicing was only observed under reducing conditions i.e. after the addition of 5 mM DTT. We propose that this is due to the requirement of a free cysteine in the reactive center of DnaE intein.

**Figure 7:**
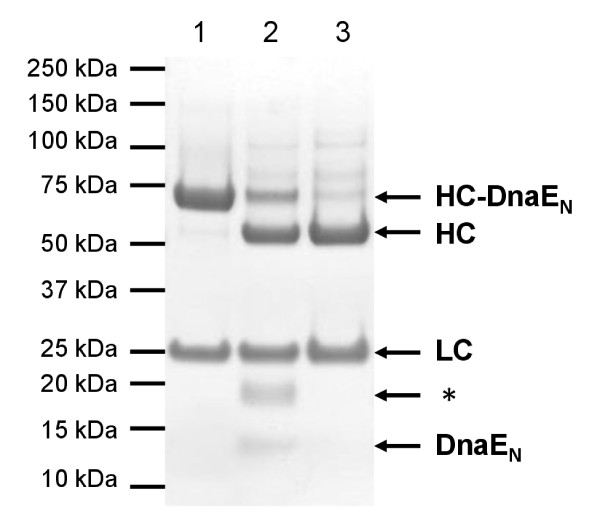
**L19 IgG-DnaE_N _after PTS**. Analysis by reducing SDS-PAGE (Coomassie stained). Purified fusion protein was treated with 5 mM DTT and 50 μM DnaE_C _peptide for 22h at RT. After dialysis, trans-spliced L19 IgG was purified by His tag affinity chromatography (1: L19 IgG-DnaE_N _before PTS, 2: after PTS, before purification, 3: after purification, HC: heavy chain, LC: light chain, * product not further investigated).

**Figure 8:**
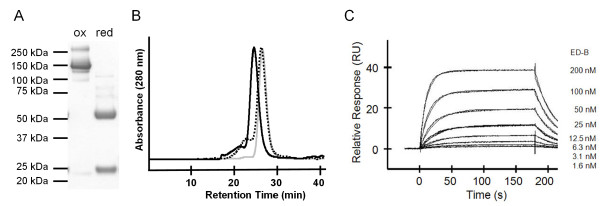
**Analysis of L19 IgG-DnaE_N _integrity after PTS**. (A) Non-reducing (ox) and reducing (red) SDS-PAGE (Coomassie stained). (B) Analytical Gel Filtration. Black: L19 IgG-DnaE_N _before PTS. Dotted: Purified PTS product. Grey: L19 IgG control antibody. (C) SPR analysis. Results were fitted to a one-site-binding model with a K_D _of 83.4 nM.

To establish the PTS protocol, DnaE_C _peptide without a probe was used. After 24h trans-splicing reaction, DTT was removed by dialysis and completely spliced L19 IgG was purified by His tag affinity chromatography (Figure 7). The yield of L19 IgG processed on both heavy chains was about 80%. Analysis were undertaken to test if the antibody had been damaged by the reducing conditions used during PTS. SDS-PAGE under non-reducing conditions showed that interchain disulfide bonds were intact as a main band at 150 kDa (calculated: 146.9 kDa) was observed (Figure 8A). However, a weak band corresponding to a protein species with an apparent molecular mass of 300 kDa indicated the presence of a small amount of antibody dimers. These results were confirmed by analytical gel filtration (Figure 8B). L19 IgG-DnaE_N _trans-splicing product eluted in a main peak very similar to unmodified L19 IgG (control antibody) but is accompanied by a small preceding peak typical for dimerization. We conclude that the integrity of L19 IgG is retained after PTS. The small percentage of dimerization could be due to some degree of hydrolysis during intein catalyzed trans-splicing (see below). This would result in antibodies with single cysteine residues at the C-terminus which are prone to pairing by disulfide bond formation. To test the functionality of L19 IgG after PTS, its interaction with the ED-B domain was analyzed by SPR. Binding kinetics and the dissociation constant (~90 nM) were the same as for the L19 IgG control antibody (Figure 8C). In addition, the interaction with the high affinity Fcγ RI/CD64 and with the low affinity Fcγ RIIIA/CD16a was investigated by SPR. Binding kinetics and the dissociation constants for both Fcγ receptors (Fcγ RI/CD64: ~0.2 nM; Fcγ RIIIA/CD16a: ~60nM) were not compromised by PTS, demonstrating the full functionality of the Fc domain (see Additional file 1). In summary, our DnaE PTS protocol does neither affect the integrity nor the functionality of L19 IgG.

**Figure 9:**
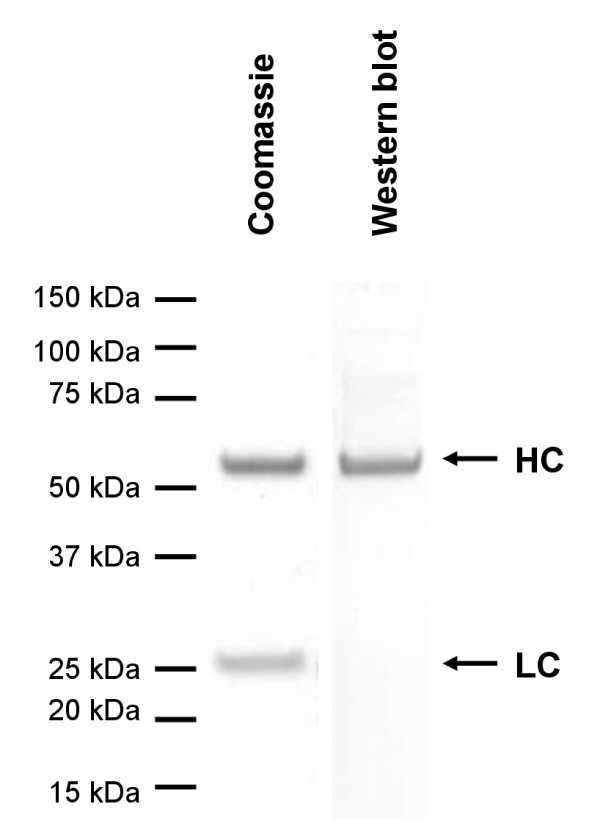
**Biotinylation of L19 IgG-DnaE_N _by PTS**. Trans-splicing was initiated with 50 μM DnaE_C_-CFNK-Biotin in the presence of 5 mM DTT. After purification by His tag affinity chromatography, the product was analyzed by reducing SDS-PAGE and Western blotting. Staining with Streptavidin-Alkaline-Phosphatase revealed specific biotin derivatization of the heavy chain.

In the next step we used the DnaE intein to attach biotin probes to the antibody. A functionalized DnaE_C _peptide (DnaE_C_-CFNK-Biotin) was applied according to our protocol and the L19 IgG product was analyzed by SDS-PAGE and Western blotting (Figure 9). Biotin was specifically attached to the heavy chain.

**Figure 10:**
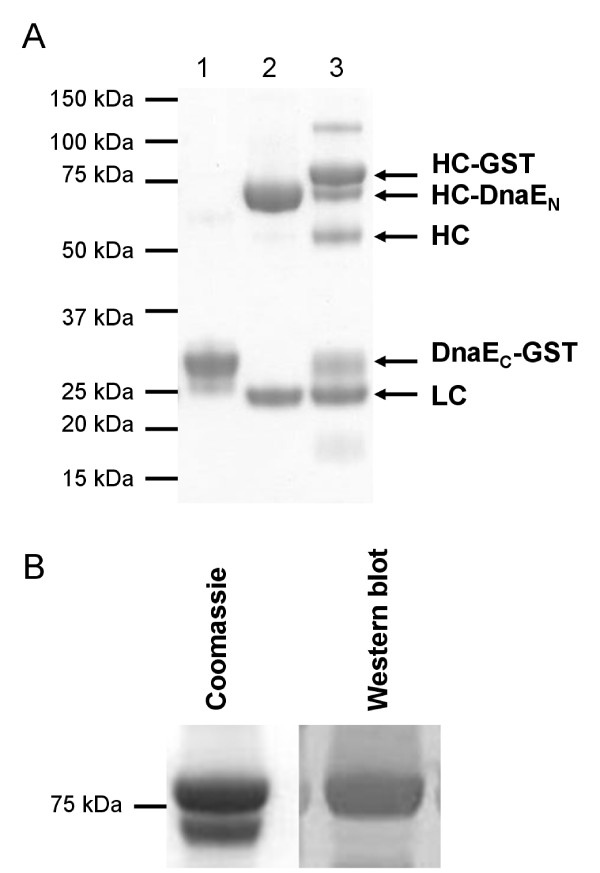
**GST transfer to L19 IgG-DnaE_N _by PTS**. Intein activation was performed with 5 mM DTT and 50 μM DnaE_C_-GST and analyzed by reducing SDS-PAGE. (A) Coomassie staining revealed HC-GST (~80 kDa) as main product and HC as side product. 1: DnaE_C_-GST. 2: L19 IgG-DnaE_N_. 3: L19 IgG-DnaE_N _+ DnaE_C_-GST. (B) Western blotting with anti-GST antibody confirmed the transfer of GST onto the heavy chain of L19 IgG (HC: heavy chain, LC: light chain).

The requirement to synthesize a 39 residue peptide (DnaE_C_-CFN) makes the described protocols for attachment of small probes more complicated with DnaE than with GyrA. However, as DnaE_C _begins on a methionine residue, it can easily be expressed in vivo at the N-terminus of a fusion protein. Proteins like exotoxins, RNases or cytokines have been described to be effective against tumor cells if fused to a targeting antibody [35]. In contrast to IgGs, these effector proteins can usually be produced by bacterial expression systems. We suggest that DnaE catalyzed PTS could be used to combine a given antibody with a diversity of effector proteins. To test our protocol for this purpose, DnaE_C _fused to glutathione-S-transferase (GST) was purified from recombinant *E. coli *and incubated with L19 IgG-DnaE_N_. Analysis by reducing SDS-PAGE showed that a main product of 80 kDa had been formed (Figure 10A). This corresponds to the heavy chain fused to GST (calculated: 76.8 kDa). Western blotting confirmed the presence of GST in the band (Figure 10B). In summary, our DnaE PTS protocol is suitable to obtain L19 IgG antibody functionalized with probes or effector proteins in good yield.

L19 IgG heavy chain conjugated to GST (80 kDa) was the main product after PTS with DnaE_C_-GST as probe. However, also a side product of 50 kDa was observed. It corresponded to the heavy chain of L19 IgG produced by hydrolysis of the thioester intermediate formed during intein catalysis and allowed evaluation of its magnitude compared to probe conjugation. We estimate from the gel that about 25% of processed heavy chain was hydrolyzed while about 75% encompassed the desired conjugation product. Due to this hydrolysis, the drug load of immunoconjugates prepared by our DnaE PTS protocol reached only about 1.5. It is likely that hydrolysis was promoted by attack of the thiol reagent DTT on the intein-derived thioester [33]. DTT had to be added to ensure reducing conditions for optimal intein activity. Alternative reducing agents like tris(2-carboxyethyl)phosphine (TCEP) could decrease hydrolysis and further optimize the PTS protocol.

## Conclusions

We have shown here for the first time that full-length IgGs can be specifically functionalized at the C-terminus of their heavy chains by EPL and PTS. Antibodies processed on both chains were obtained in good yields and purified from incompletely processed proteins. The attachment of two probes per antibody and in vicinity to each other yielded an additional disulfide bond, circumventing problems with free thiol groups introduced by intein-fusion technologies. These methods could be employed to generate immunoconjugates of high homogeneity and with a drug load of two. The modification at the C-termini of the heavy chains does not interfere with antigen binding and, judged by binding to Fcγ RI and Fcγ RIIIA, it does not compromise the effector functions of the Fc domain. Provided with an effective toxophore, this would result in therapeutic antibodies fully equipped for the elimination of malignant cells.

## Methods

### General

Chemicals were purchased from Sigma Aldrich if not stated otherwise. Chromatography material (Glutathione Sepharose 4 Fast Flow, HiTrap Chelating HP, Superdex 200) was obtained from GE Healthcare (Munich).

### Vectors

L19 IgG was derived by transferring the variable domains of L19 scFv [36] into a human IgG1/к format.

*Mxe *GyrA (from vector pTXB1, New England Biolabs) or *Npu *DnaE_N _(from vector pSKDuet, provided by Hideo Iwai, University of Helsinki) were cloned behind the heavy chain of L19 IgG, separated by a linker coding for GLEGGSGGSEY. GyrA and DnaE_N _were extended C-terminally to include a His tag (GSA-H_10 _and AS-H_10_, respectively). Constructs encoding L19 IgG (control, without linker) and L19 IgG fused to GyrA or DnaE_N _were transferred into pTT5 vector (National Research Council Canada). In the resulting expression vectors, both, heavy and light chain, were under the control of an hCMV promoter.

DNA encoding DnaE_C _from *Nostoc punctiforme *(MIKIATRKYLGKQNVYDIGVERDHNFALKNGFIASN [32]), followed by a linker (CFNPAGSSGVIM) and Glutathione-S-Transferase (GST, *Schistosoma japonicum*) was purchased from Geneart (Regensburg, Germany), amplified by PCR and ligated into vector pET29a (Novagen) by NdeI/NotI.

### Expression and Purification of L19 IgG constructs

0.5 L of HEK293-6E cells were grown in F17 medium with 4 mM GlutaMAX-I and 0.1% Pluronic F-68 (Invitrogen) to a density of 1.5 - 2.0 × 10^6 ^cells/mL and transfected with 500 μg plasmid DNA (L19 IgG constructs) as described [37], with minor modifications. After five days cells were harvested by centrifugation (8000 × g, 5'). The supernatant was concentrated to 50 mL by crossflow filtration (Sartorius) and dialyzed against column buffer (500 mM NaCl, 50 mM Hepes-NaOH, pH 7.5). For purification, Ni-loaded IMAC (elution with imidazole gradient) and subsequent gel filtration was applied.

### Expression and Purification of DnaE_C_-GST

*E. coli *BL21-Star (DE3) (Invitrogen) was transformed with vector DnaE_C_-GST-pET29a. Medium 2xYT (Difco) containing 50 μg/mL kanamycin was inoculated with an overnight culture and grown at 37°C, 220 rpm. At an OD_600 _of 0.4 temperature was decreased to 16°C. Protein expression was induced with 0.3 mM IPTG at an OD_600 _of 0.8. After 48 h cells were harvested (3,000 × g, 20') and suspended in buffer (500 mM NaCl, 50 mM Tris-HCl pH 7.5, 2 mM β-mercaptoethanol). Cell lysate was obtained by high pressure homogenization (Constant cell disruption systems, Constant Systems Ltd, UK) and subsequent centrifugation (20,000 × g, 30'). Purification of DnaE_C_-GST protein was achieved by applying Glutathione Sepharose (500 mM NaCl, 50 mM Tris-HCl pH 7.5, 2 mM β-mercaptoethanol, elution with 10 mM reduced glutathione), followed by gel filtration (500 mM NaCl, 50 mM Hepes-NaOH, pH 7.5).

### Intein catalyzed protein modification

For GyrA mediated EPL, 5 μM L19 IgG-GyrA was incubated in 500 mM NaCl, 50 mM Hepes-NaOH, pH 8.0, 50 mM Mesna (2-mercaptoethane sulfonate-sodium), 5 mM cysteine or Bio-P1 Peptide (NEB). For DnaE mediated protein trans-splicing, 5 μM L19 IgG-DnaE_N _was mixed with 50 μM DnaE_C _Peptide (MVKVIGRRSLGVQRIFDIGLPQDHNFLLANGAIAANCFN, kind gift from Prof. Christian Becker, TU Munich, Germany. The sequence corresponds to DnaE_C _from *Synechocystis sp*. strain PCC6803 (*Ssp*) which is interchangeable with *Npu *DnaE_C _[32]), DnaE_C_-Biotin (MVKVIGRRSLGVQRIFDIGLPQDHNFLLANGAIAANCFNK-Biotin, synthesis by JPT Peptide Technologies GmbH, Berlin, Germany) or DnaE_C_-GST in 500 mM NaCl, 50 mM Hepes-NaOH, pH 7.5, 5 mM DTT. After incubation for 22-24h at room temperature (RT), intein activity was stopped by removing reducing agents (Mesna, DTT, cysteine) by dialysis over night at RT. For purification, the sample was loaded onto a Ni-IMAC column (500 mM NaCl, 50 mM Hepes-NaOH, pH 7.5). Completely processed L19 IgG was not in the flow through but was eluted at 30 mM imidazole. Partly- or non-processed L19 IgG-GyrA or -DnaE_N _was eluted with 300 mM imidazole. For subsequent analysis, protein was dialyzed against 500 mM NaCl, 50 mM Tris-HCl, pH 7.5 and concentrated to 1-2 mg/mL.

### SEC

A sample (~50 μg) was loaded onto a gel filtration column connected to an HPLC instrument (Agilent Technologies 1200 Series) equipped with detectors for refractive indices (RI) and multi angle light scattering (MALS) (Optilab rEX and miniDAWN TREOS, Wyatt Technology Corp., Santa Barbara, CA). To calculate the yield of intein processing, a sample after EPL/PTS but before purification was analyzed. The mass of the excised intein was determined by its RI signal and set into relation to the total loaded mass.

### Pepsin digestion and mass spectrometry

L19 IgG was dialyzed against 20 mM NaAcetat pH 4.5. Pepsin (Protea Biosciences) was added to give an enzyme-to-antibody ratio of 1:25 (w:w) and incubated for 24h at 37°C. A sample was directly analyzed by ESI-TOF to assess masses of peptides derived by the Pepsin digest. Further samples were separated by reducing and non-reducing SDS-PAGE and gels were Coomassie stained. Protein bands were excised, de-stained, reduced with DTT, alkylated with iodacetamide and digested with trypsine. Protein sequences were determined by ESI-Q-TOF (MS and MS/MS) and search in Sequest (Finnigan) and Mascot Databases.

### Western blot

After SDS-PAGE, gels were blotted onto a nitrocellulose membrane (iBlot, Invitrogen) and incubated for 1h in blocking buffer (TBS with 0.05% (v/v) Tween-20 and 5% (w/v) milk powder). Biotin was detected by incubating with 1 μg/ml Streptavidin-Alkaline Phosphatase (Sigma) in blocking buffer over night. GST was detected by over night incubation with anti-GST antibody (GE Healthcare, 1:5000 in blocking buffer) followed by 1h incubation with anti-Goat IgG-Alkaline Phosphatase (Sigma, 1:1000 in blocking buffer) as secondary antibody. BCIP/NBT (SigmaFAST tablet) was used for membrane staining.

### Surface Plasmon resonance (SPR)

A Biacore T100 instrument (GE Healthcare, Munich) was employed to study the interaction between L19 IgG or modified versions thereof and ED-B, Fcγ RI/CD64 and Fcγ RIIIA/CD16a. L19 IgG or modified versions thereof were immobilized on a Biacore Sensor Chip (Series S Sensor Chip CM5 treated with antibody capture kit, GE Healthcare). Soluble ED-B domain in several concentrations (1.56 - 200 nM) or soluble Fc receptor fragments Fcγ RI/CD64 and Fcγ RIIIA/CD16a (R&D Systems) in several concentrations (1.56 - 50 nM) were used as analytes. Association and dissociation kinetics were detected to calculate the dissociation constant.

## Competing interests

The authors are employees of Bayer Healthcare and declare that they have no competing interests.

## Authors' contributions

SM established the in vitro cleavage protocols, carried out protein expression, purification and analysis and wrote the manuscript. SG carried out the tryptic digest and the mass spectrometry. PB participated in the conceptional design of this project. AH supervised the research and contributed to the writing of the manuscript. All authors read and approved the final manuscript.

## Supplementary Material

Additional file 1 **Table S1**. Test of Fc functionality after EPL and PTS. The interaction with two Fcγ receptors was analyzed by SPR. Click here for file
