# Supplementary material for: Site-specific modification of ED-B-targeting antibody using intein-fusion technology
Source: BMC Biotechnol. 2011 Jul 21;11:76. doi: 10.1186/1472-6750-11-76 (PMC3154154; doi:10.1186/1472-6750-11-76)
Supplement: Additional file 1 — Table S1. Test of Fc functionality after EPL and PTS. The interaction with two Fcγ receptors was analyzed by SPR. [file 1472-6750-11-76-S1.PDF]

Table S1. Test of Fc functionality after EPL and PTS. The interaction with two Fcγ receptors was analyzed by SPR.

L19 IgG	Fcγ RI/CD64			Fcγ RIIIA/CD16a		
	k_{on} [$M^{-1}s^{-1}$]	k_{off} [s^{-1}]	K_d (nM)	k_{on} [$M^{-1}s^{-1}$]	k_{off} [s^{-1}]	K_d (nM)
control antibody	1.02×10^{6}	1.68×10^{-4}	0.17	1.16×10^{6}	1.03×10^{-1}	89
L19 IgG-DnaE$_N$ after PTS	0.96×10^{6}	2.01×10^{-4}	0.21	0.72×10^{6}	0.40×10^{-1}	56
L19 IgG-GyrA after EPL	1.11×10^{6}	1.45×10^{-4}	0.13	1.67×10^{6}	0.60×10^{-1}	36
